# Supplementary material for: The Fast Evolution of the Stenobothrini Grasshoppers (Orthoptera, Acrididae, and Gomphocerinae) Revealed by an Analysis of the Control Region of mtDNA, with an Emphasis on the Stenobothrus eurasius Group
Source: Insects. 2024 Aug 3;15(8):592. doi: 10.3390/insects15080592 (PMC11354746; doi:10.3390/insects15080592)
Supplement: Supplementary file 1 [file insects-15-00592-s001.zip › insects-3043595-supplementary.pdf]

Table S1. List of the Gomphocerinae species and NCBI nucleotide/**BOLD** accession numbers of sequences used in phylogenetic analysis.

Species	COI			control region		
	Accession №	Sample locality	Reference	Accession №	Sample locality and reference	Reference
Chorthippus pullus	ON828416	Ukraine, Cherkassy region, Cherkassy district, Kanev	[17]	PP239129	Russia, Ulyanovsk region, Novospasskoe district, Monastyrsky Sungur	This study
Euchorthippus unicolor	-	-		MK113716	China, Xi'an	[70]
Gomphocerripus rufus	-	-		MK903592	China, Inner Mongolia	[23]
Myrmeleotettix antennatus	PP177431	Russia, Saratov region, Krasnokutsk district, D'jakovka		PP239130	Russia, Ulyanovsk region, Nikolaevsk district, Kanadey	This study
	HQ738954	Ukraine, Kherson region Henichesk district, Ivanivka	[11]	PP239131		
Myrmeleotettix maculatus	HQ738955	Ukraine, Nikolaev region Arbusinka district, Ostapovka	[11]	PP239132	Russia, Ulyanovsk region, Novospasskoe district, Monastyrsky Sungur	This study
	JN167822	Poland, Bledwowska desert	[71]			
	KC480253	Spain, western Cantabrian Mountains	[72]			
	MH106818	Germany, Bavaria, Lower Bavaria, Garmisch-Partenkirchen district, Ettal	[73]			
Myrmeleotettix pallidus	PP177426	Kazakhstan, Pavlodar region, Irtyshsk district, Irtyshsk	This study	-	-	
	PP190403	Kazakhstan, Akmola region, Yereymentau district, Baysary	This study			
Myrmeleotettix palpalis	PP190404	Russia, Altai republic, Ongudai district, Kupchegen	This study	PP239133	Russia, Altai republic, Ongudai district, Kupchegen	This study
	ON422106	China, Gansu prov., Gannan Tibetan	Unpublished	MK903595	China, Gansu prov., Sunan	[23]

		Autonomous Pref., Xiahe County, Qiayi				
	ON422105<br>ON426221<br>ON426222<br>ON426223	China, Gansu, Zhangye, Shandan County, Malianjing	Unpublished			
Omocestus haemorrhoidalis	KC140082	China, Shaanxi, Huayin	[74]	PP239134	Kazakhstan, Jetisu region, Kerbulak district, Altyn Emel pass	This study
	MK903570	China, Shaanxi, Huayin	[23]			
Omocestus minutus	PP177432	Russia, Saratov region, Krasnokutsky district, D'jakovka	This study	-	-	
	HQ738956	Ukraine, Odessa region	[11]			
Omocestus panteli	DQ230728	Spain, Sierra Nevada	[53]	-	-	
	MT311123	Spain, Cantabrian Mountains	[76]			
Omocestus petraeus	-	-		MK903596	China, Inner Mongolia, Erzhona	[23]
Omocestus rufipes	HQ738957	Greece, N. Kerkyra	[11]	-	-	
	JN187509	France, Bourgogne, Saône-et-Loire, Vendenesse-sur-Arroux	[53]			
Omocestus viridulus	GU706158	Germany, Bavaria, Lower Bavaria, Regen district, Achslach	[18]	PP239135	Russia, Altai republic, Ongudai district, Seminsky pass	This study
	HQ738958	Russia, Leningrad region	[11]			
	JN299382	Germany, Bavaria, Upper Bavaria, Munich	[18]			
	KC480249	Spain, western Cantabrian Mountains	[72]			
	AY738363	Russia, Altai republic	[73]			
Pseudochorthippus parallelus	MT166300	Spain	[28]	MT166298	Spain	[28]
Stenobothrus carbonarius	-	-		PP239136<br>PP239137	Kazakhstan, Almaty region, Kegen district, Kegen	This study
Stenobothrus cotticus	FJ555552<br>FJ555553	Bulgaria	[39]	-	-	
	FJ555555	France, Hautes-Alpes, Col d'Izoard	[39]			
Stenobothrus eurasius (allopatric populations)	KM816669<br>KM816671<br>KM816673	China, Xinjiang	[78]	PP239138<br>PP239139<br>PP239140	Russia, Altai Krai, Barnaul district, Barnaul	This study
	**GBORT800-15**	Austria, Lower Austria	[18]	PP239141		

	**GBORT801-15**			PP239142		
	**GBORT802-15**			PP239143		
	**GBORT803-15**			PP239144		
	HQ738961	Ukraine, Kherson region, Kherson district, Oleshki	[11]	PP239145		
	AY738366	Russia, Altai republic	[77]	PP239146	Russia, Altai republic, Ongudai district, Ongudai	This study
	OR454364	Hungary, Belapatfalva, Belko	[41]	PP239160		
	OR454365			PP239161		
	OR454366			PP239162		
	OR454367	Hungary, Budaors, Odvas-hegy	[41]	PP239163		
	OR454368			PP239164		
	OR454369			PP239165		
	OR454370			PP239166		
	OR454373	Romania, Cerna, Munții Macinului	[41]	PP239151	Russia, Altai republic, Ongudai district, Kupchegen	This study
	OR454374			PP239152		
	OR454375			PP239153		
	OR454376	Hungary, Füzer, Kopaszka	[41]	PP239154	Russia, Altai republic, Kosh-Agach district, Kyzyl-Tash	This study
	OR454377	Romania, Greci, Munții Macinului	[41]	PP239155		
	OR454378	Austria, Hainburg, Braunsberg	[41]	PP239156		
	OR454381	Romania, Izovoarele, Dealul Consul	[41]	PP239157		
	OR454382					
	OR454383					
	OR454388	Czech Republic, Mnichov, Oblik	[41]			
	OR454389					
	OR454390					
	OR454402	Czech Republic, Rana, Rana	[41]			
	OR454403					
	OR454405	Hungary, Szarliget, Nagy-Szena-hegy	[41]			
	OR454406					
	OR454408					
	OR454409					
	OR454410	Hungary, Tokaj, Kopasz-hegy	[41]			
	OR454411	Hungary, Tes, Ber-hegy	[41]			
	OR454412					
	FJ555547	Bulgaria	[39]			

Stenobothrus eurasius (contact zone)	PP190405	Russia, Saratov region, Saratov	This study	PP239147	Russia, Volgograd region, Mikhaylovka district, Ilmensky 2-y khutor	This study
	HQ738960	Russia, Saratov region, Saratov	[11]	PP239148	Russia, Ulyanovsk region, Nikolaevsk district, Kanadey	This study
				PP239149		
				PP239150		
				PP239158	Russia, Ulyanovsk region, Novospasskoe district, Monastyrsky Sungur	This study
				PP239159		
				PP239167	Russia, Samara region, Syzran' district, Staraya Racheyka	This study
				PP239168		
				PP239169	Russia, Saratov region, Saratov	This study
Stenobothrus festivus	MW286134	Spain, Asturias	[72]	-	-	
Stenobothrus fischeri	PP177433	Russia, Volgograd region, Volgograd, Sarepta	This study	PP239170	Kazakhstan, Jetisu region, Aksu district, Kapal	This study
	PP177434			PP239171		
	PP177435					
	PP177427					
	ON828421	Russia, Volgograd region, Volgograd, Sarepta	[17]			
	HQ738962	Greece: Ipiros, Ioannina, Timfi-Mt., Vikos Park	[11]			
	FJ555548	Greece	[39]			
Stenobothrus hyalosuperficies (allopatric populations)	ON828422	Kazakhstan, Almaty region, Kerbulak district, Altyn Emel pass	[17]	PP239172	Kazakhstan, Almaty region, Kerbulak district, Altyn Emel pass	This study
				PP239173		
				PP239175	Kazakhstan, Jetisu region, Aksu district, Kapal	This study
				PP239176		
				PP239182	Kazakhstan, Jetisu region, Sarkand district, Pokatilovka	This study
				PP239183		
				PP239185	Kazakhstan, Almaty region, Raiymbek district, Temirlik	This study
				PP239186		
Stenobothrus hyalosuperficies (contact zone)	PP190406	Russia, Saratov region, Ershov district, Ershov	This study	PP239174	Russia, Samara region, Syzran' district, Kashpir	This study
				PP239177	Russia, Saratov region, Saratov	This study
				PP239178	Russia, Saratov region, Vol'sk district, Shiroky Buerak	This study
				PP239179		
				PP239180		
				PP239181		
				PP239184	Russia, Volgograd region, Kamyshinsky district, Shcherbakovka	This study
				PP239187		This study

				PP239188<br>PP239189<br>PP239190	Russia, Volgograd region, Kamyshinsky district, Nizhnyaya Dobrinka	
Stenobothrus lineatus	**DTNHM5559-23**	United Kingdom, England	[18]	PP239191	Russia, Ulyanovsk region, Nikolaevsk district, Kanadey	This study
	**FBORT380-10**	Germany, Bavaria, Upper Bavaria	[18]	PP239192		
	**FBORT512-13**	Germany, Bavaria, Upper Palatinate	[18]			
	**FBORT535-13**					
	**GBMIX3289-16**	Switzerland, Grisons canton, Maloja region, Madulain	[18]			
	**GBMIX3306-16**	Germany, Rhineland-Palatinate, eastern Doerscheid	[18]			
	GU706142<br>GU706143	Germany, Bavaria, Amberg-Sulzbach, Ransbach	[18]			
	GU706145	Germany, Bavaria, Upper Bavaria, Freising district, Neufahrn by Freising	[18]			
	HQ738963	Russia, Moscow region, Prioksko-Terrasny reserve	[11]			
	FJ555549	Bulgaria	[39]			
	MT011531	Germany, Lower Saxony, Lüchow-Dannenberg, Höhbeck	[79]			
	MT311128	Spain, Cantabrian Mountains	[76]			
Stenobothrus miramae	HQ738964	Ukraine, Crimea [11]	This study	PP239193<br>PP239194	Russia, Saratov region, Krasnoarmeysk district, Belogorskoe	This study
Stenobothrus newskii	PP190407	Russia, Altai republic, Ulagan district, Lake Cheybekkel	This study	PP239195	Russia, Altai republic, Ulagan district, Aktash	This study
	PP190408			PP239196		
Stenobothrus nigromaculatus	**FBORT517-13**	Germany, Bavaria, Upper Palatinate	[18]	PP239197	Russia, Saratov region, Vol'sk district, Shiroky Buerak	This study
	**FBORT526-13**			PP239198		
	**FBORT528-13**					

	**FBORT529-13**					
	**FBORT530-13**					
	**FBORT532-13**					
	KC480252	Spain, western Cantabrian Mountains	[72]			
	GU706144	Germany, Bavaria, Upper Bavaria, Freising district, Neufahrn by Freising	[18]			
	GU706102					
	HQ738965	Moldova, Bolshaya Khalakhora	[11]			
	JN187510	France, Provence-Alpes-Côte d'Azur, Alpes-Maritimes, Valdeblore	[53]			
	OR454371	Hungary, Csor, Szaraz-horog-völgy	41]			
	OR454372					
	OR454379	Czech Republic, Horenec, Cicov [				
	OR454380					
	OR454384	Hungary, Labatlan, Pisznice	[41]			
	OR454385	Czech Republic, Miroslav, Miroslavske kopce	[41]			
	OR454386					
	OR454391	Slovakia, Nitra, Zobor Hill	[41]			
	OR454392					
	OR454393					
	OR454394					
	OR454395	Hungary, Perbal, Meszes-hegy	[41]			
	OR454396					
	OR454397					
	OR454398					
	OR454399					
	OR454400					
	OR454401					
	OR454404	Czech Republic, Rana, Rana	[41]			
	OR454413	Hungary, Tes, Ber-hegy	[41]			
	OR454414					
Stenobothrus rubicundulus	**GBORT983-15**	Slovenia, Slovene Istria	[18]	-	-	
	HQ738966	Greece, Ipiros, Ioannina	[11]			

	FJ555551	Bulgaria	[39]			
Stenobothrus stigmaticus	HQ738967	Germany, Hesse	[11]	-	-	
	JN187511	France, Bourgogne, Saône-et-Loire, Vendenesse-sur-Arroux	[53]			
Stenobothrus sviridenkoi	AY738365	Armenia	[77]	-	-	
